# Sequential cryoglobulinemia-associated glomerulonephritis and light-chain amyloidosis in monoclonal gammopathy of renal significance

**DOI:** 10.1007/s13730-026-01108-3

**Published:** 2026-04-07

**Authors:** Yuki Nakayama, Makoto Harada, Koji Hashimoto, Takashi Ehara, Yuji Kamijo

**Affiliations:** 1 Department of Nephrology, Shinshu University School of Medicine, 3-1-1, Asahi, Matsumoto, 390-8621 Japan; 2 Nagano Kidney Evolution Association, 2-17-5, Tsukama, Matsumoto, Japan

**Keywords:** AL amyloidosis, Cryoglobulinemic membranoproliferative glomerulonephritis, Kidney biopsy, Monoclonal gammopathy of renal significance

## Abstract

Kidney disorders associated with monoclonal gammopathy are diagnostically challenging because diverse kidney lesions can occur during the disease course. Here, we report a man in his early 60s who presented with edema, proteinuria, and impaired kidney function. Kidney biopsy revealed cryoglobulinemia-associated glomerulonephritis. Although bone marrow examination showed no increase in plasma cells, the patient was diagnosed with monoclonal gammopathy of renal significance (MGRS) and treated with steroids and cryofiltration, resulting in transient improvement. Later, proteinuria worsened, and kidney function declined despite intensified steroid therapy. A repeat kidney biopsy demonstrated AL (lambda) amyloid deposition in the glomeruli, leading to a diagnosis of AL amyloidosis. This case illustrates that distinct kidney pathologies can emerge sequentially in patients with MGRS and underscores the importance of repeat kidney biopsies when clinical findings change.

## Introduction

Cryoglobulinemia is characterized by the presence of immunoglobulins that precipitate at low temperatures and can lead to a membranoproliferative glomerulonephritis (MPGN) pattern via immune complex deposition in the kidneys [1]. AL amyloidosis is a rare disorder caused by the deposition of monoclonal immunoglobulin light chains, leading to progressive organ dysfunction that commonly affects the kidneys and heart, with fatal outcomes [2]. Although both conditions result from abnormal levels of circulating proteins, their clinical manifestations and histopathological features differ significantly. Cryoglobulinemia and AL amyloidosis are manifestations of underlying hematological abnormalities. Recently, attention has been focused on disorders termed monoclonal gammopathy of renal significance (MGRS), in which kidney involvement occurs in the absence of criteria for initiating therapy for the underlying hematological condition [3].

Here, we report a rare case of a patient initially diagnosed with cryoglobulinemia-associated glomerulonephritis who was newly diagnosed with AL amyloidosis in the kidney. Despite the absence of increased plasma cells in the bone marrow, continued monoclonal gammopathy was thought to be related to kidney disease (MGRS), and the patient exhibited two distinct forms of MGRS. This case underscores the diagnostic complexity of kidney disorders associated with monoclonal gammopathy and highlights the fact that different forms of MGRS may be present even after a prolonged clinical course.

## Case report

A man in his early 60s presented to our hospital for the evaluation of edema, proteinuria, and kidney dysfunction. On admission, his body temperature was 36.2 °C, blood pressure was 143/90 mmHg, and heart rate was 52 bpm. Physical examination revealed no obvious abnormalities, except bilateral lower leg edema. Laboratory findings (Table 1) revealed anemia (hemoglobin 8.1 g/dL), hypoalbuminemia (2.8 g/dL), proteinuria (4.11 g/gCre), and kidney dysfunction (Serum creatinine 1.18 mg/dL). He also had hypocomplementemia and tested positive for cryoglobulin; however, antinuclear antibodies, anti-double-stranded DNA antibodies, and rheumatoid factor were negative. Cryoglobulin analysis identified monoclonal IgG (lambda) and polyclonal IgG (kappa with lambda), leading to the diagnosis of type 2 cryoglobulinemia. Serum-free light chain analysis revealed a lambda dominance. Bone marrow biopsy revealed no increase in plasma cells (2.8%). Active infection with hepatitis B and/or C viruses was not observed, and no malignancy was detected on computed tomography or gastrointestinal endoscopy. A kidney biopsy was performed to evaluate the cause of renal dysfunction and massive proteinuria.

**Table 1:** Laboratory data of the first and second kidney biopsies

	At first kidney biopsy	At the second kidney biopsy	Normal range
*Urine*			
Red blood cell sediment (/HPF)	1–4	5–9	< 5
Protein (g/gCre)	4.11	4.49	< 0.15
*Blood*			
White blood cell (10^3^/μL)	4.60	6.53	3.3–8.6
Red blood cell (10^6^/μL)	3.48	3.68	4.35–5.55
Hemoglobin (g/dL)	8.1	11.8	13.7–16.8
Platelet (× 10^4^/μL)	22.8	21.9	15.8–34.8
Total protein (g/dL)	6.0	5.0	6.6–8.1
Albumin (g/dL)	2.8	2.7	4.1–5.1
Urea nitrogen (mg/dL)	23.0	29.5	8–20
Creatinine (mg/dL)	1.18	1.25	0.63–1.05
eGFR (mL/min/1.73 m^2^)	49	44	
C-reactive protein (CRP)	0.12	0.05	< 0.14
Aspartate aminotransferase (IU/L)	19	44	13–30
Alanine transaminase (IU/L)	20	49	10–42
Lactate Dehydrogenase (IU/L)	194	246	124–222
Alkaline phosphatase (U/L)	154	151	106–322
Sodium (mmol/L)	139	141	138–145
Potassium (mmol/L)	4.6	4.6	3.6–4.8
Complement activities50 (U/mL)	< 4	41.4	30–53
Complement3 (mg/dL)	31	77	86–160
Complement4 (mg/dL)	4.1	23.9	17–45
IgG (mg/dL)	1960	875	870–1700
IgA (mg/dL)	50	37	110–410
IgM (mg/dL)	50	26	46–260
Antinuclear antibody (ANA)	negative	negative	negative
Anti-double-stranded DNA antibody (IU/mL)	3.6	NA	
Rheumatoid factor (IU/mL)	2	2	0–10
Cryoglobulin levels	10.5	5.0	
Analysis of cryoglobulins	IgG(λ) + IgG(λ + κ)	NA	
Immunofixation electrophoresis	IgG(λ)	IgG(λ)	
Free light chain κ/λ	0.129	0.240	0.248–1.804
Kappa chain (mg/L)	50.1	37.1	2.42–18.92
Lambda chain (mg/L)	389.0	151.9	4.44–26.18
HBsAg (IU/mL)	NA	0.001	< 0.004
HBsAb (mIU/mL)	14.7	14.5	0–9.9
HBcAb (COI)	0.2	0.1	0–0.9
HCVAb (COI)	0.3	0.3	0–0.9

Ab, antibody; Ag, antigen; eGFR, estimated glomerular filtration rate; IgA, Immunoglobulin A; IgG, Immunoglobulin G; IgM, Immunoglobulin M; HB, hepatitis B virus; HCV, hepatitis C virus

**Fig. 1:**
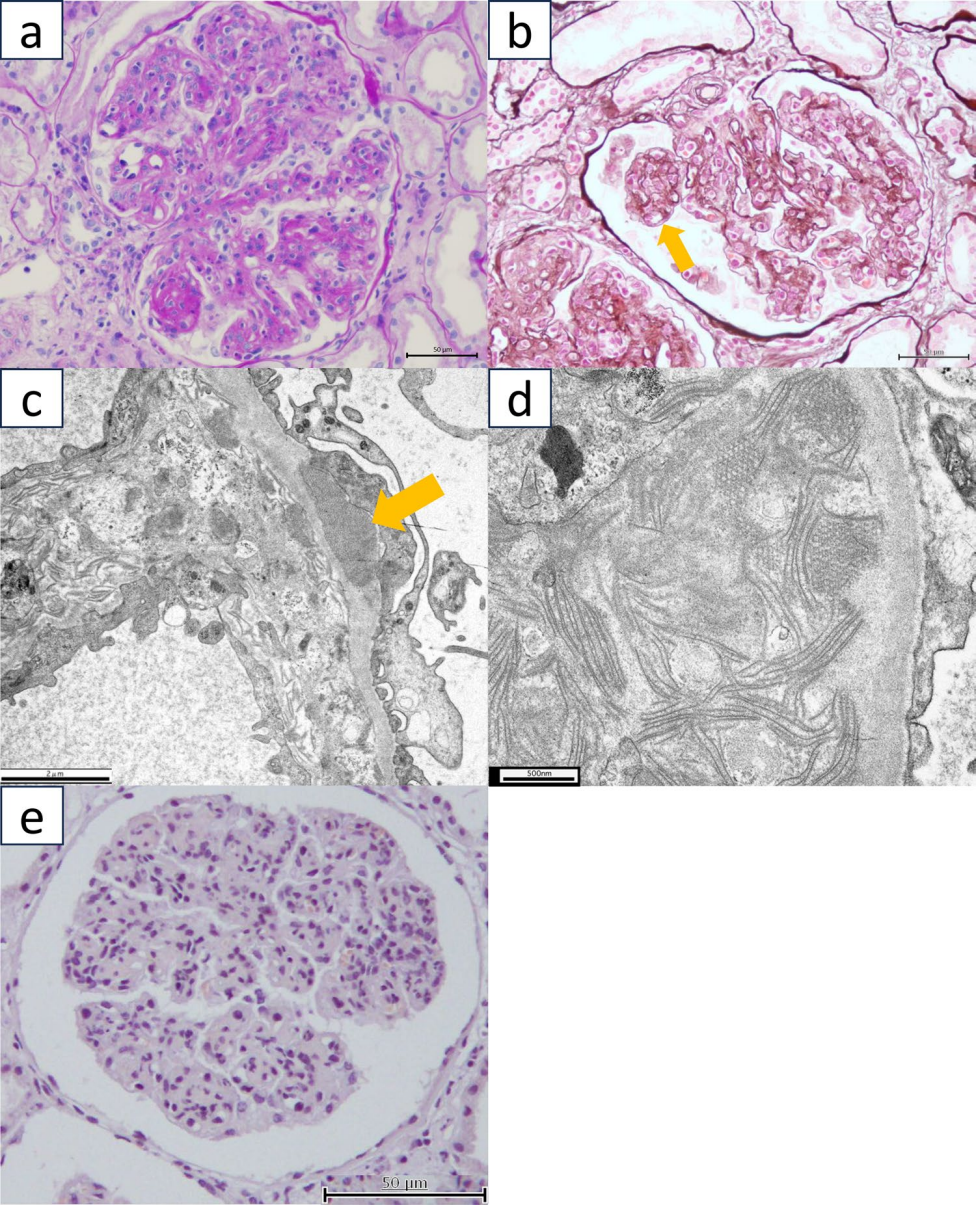
Pathological findings of a first kidney biopsy. **a** Periodic acid–Schiff (PAS) staining of the first kidney biopsy specimen revealed mesangial expansion. **b** Periodic acid methenamine silver (PAM) staining demonstrated a double-contour appearance in the first kidney biopsy specimen (arrow). **c** Electron microscopy of the first kidney biopsy specimen revealed sub-epithelial electron-dense deposits (arrow). **d** Tubular structures approximately 700 nm in length and 35 nm in diameter were identified in the first kidney biopsy specimen. **e** Congo red staining was negative in the first biopsy specimen

Light microscopy of the kidney biopsy specimen containing 33 glomeruli did not reveal global sclerosis. The glomeruli exhibited an increased mesangial matrix, mesangial cell hypercellularity, and endocapillary hypercellularity (Fig. 1a). Double contours were also observed (Fig. 1b). Immunofluorescence staining was negative for IgG, IgM, IgA, and fibrinogen but positive for C3. Kappa and lambda light chains were negative under immunofluorescence staining. Electron microscopy revealed fibrillary deposits with tubular structures in the subepithelial areas. These structures were approximately 700 nm in length and 35 nm in diameter (Fig. 1c, d). Congo red staining results were negative (Fig. 1e). Based on these results, the primary diagnosis was cryoglobulinemia-associated glomerulonephritis. Glucocorticoid therapy (prednisolone 50 mg/day) was initiated, and cryofiltration was additionally performed. The clinical course is presented as a clinical chart in Fig. 3. Blood cryoglobulin levels decreased, and both proteinuria and kidney function improved. Three years after the initial therapy, the cryoglobulin level increased, and glucocorticoid therapy was intensified. The cryoglobulin levels showed limited improvement. Eight years after the initial hospitalization, the proteinuria worsened again. Proteinuria was resistant to steroid therapy, and the cryoglobulin level remained low, which did not correspond to the increasing amount of proteinuria. A second kidney biopsy was performed 11 years after the initial hospitalization.

**Fig. 2:**
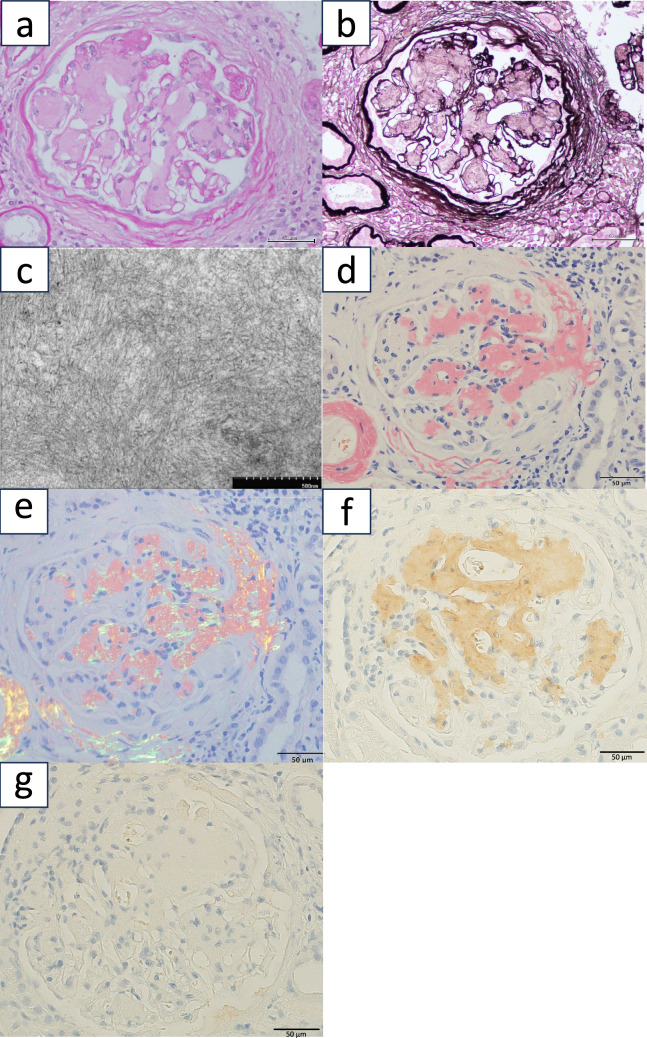
Pathological findings of a second kidney biopsy. **a** Periodic acid–Schiff (PAS) staining of the second kidney biopsy specimen. **b** Periodic acid methenamine silver (PAM) staining demonstrated nodular lesions in the second kidney biopsy specimen. **c** Electron microscopy of the second kidney biopsy specimen. **d** Congo red staining was positive in the second biopsy specimen. **e** Polarized light microscopy revealed apple green amyloid. **f** Immunostaining of the second kidney biopsy specimen revealed deposition of lambda-type amyloid light chains. **g** Immunostaining of the second kidney biopsy specimen was negative for kappa light chains

**Fig. 3:**
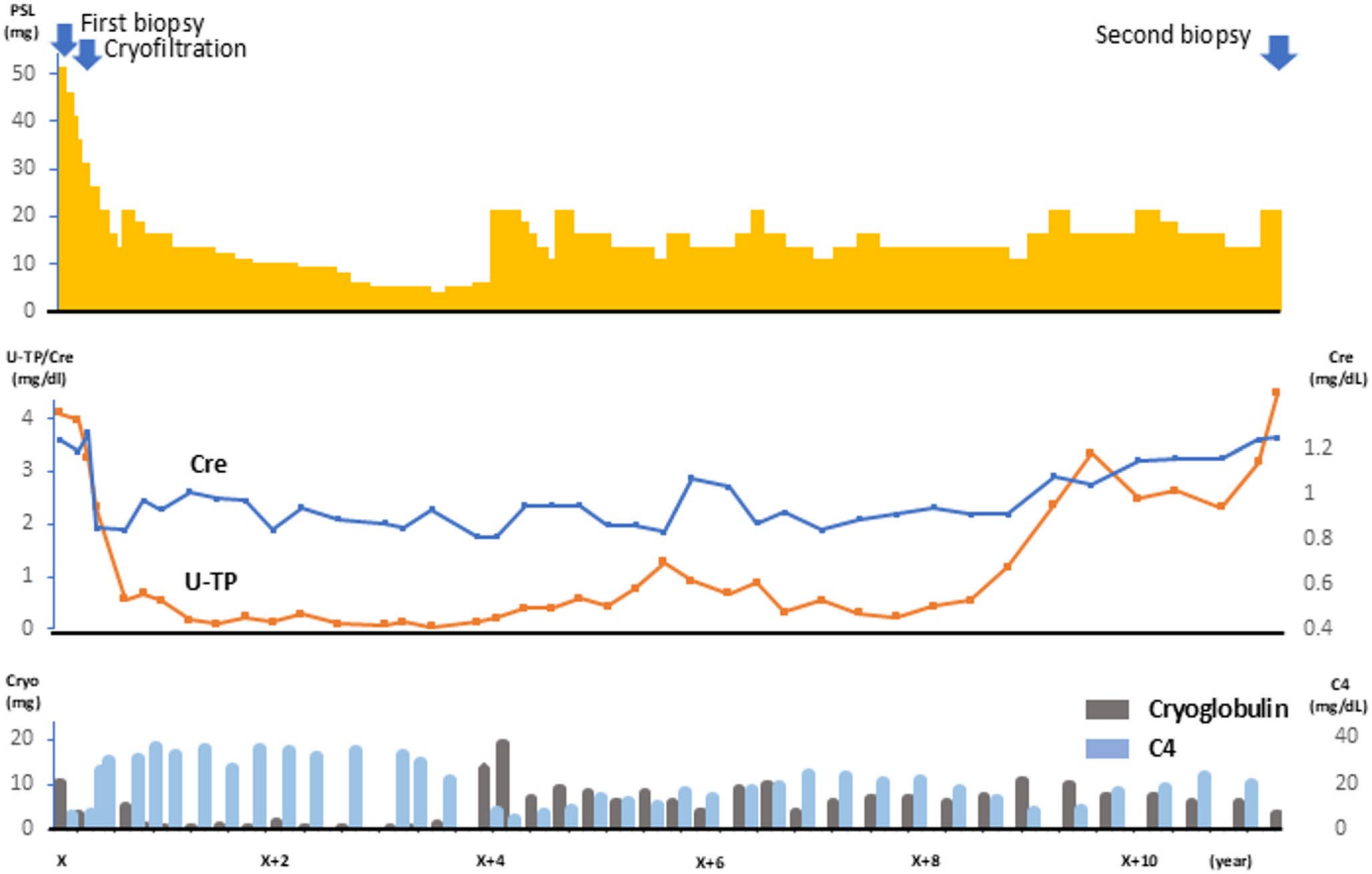
Clinical course of the current case

Light microscopy of the kidney biopsy specimen, which contained 29 glomeruli, revealed 14 global sclerosis. There was no increase in mesangial cellularity, but there was a marked mesangial expansion and nodular lesions in some glomeruli (Fig. 2a, b). Immunofluorescence staining was negative for IgG, IgM, IgA, fibrinogen, and C3. Electron microscopy revealed fibrillary deposits 10–15 nm in diameter, arranged in bundles (Fig. 2c). Congo red staining was positive, apple green birefringence was confirmed under a polarizing microscope (Fig. 2d, e), and lambda-amyloid light chains were identified by immunostaining (Fig. 2f, g). Serum immunofixation electrophoresis confirmed the presence of IgG (lambda)-type monoclonal gammopathy. Bone marrow examination showed no increase in plasma cells, which was consistent with the initial evaluation. Systematic evaluation revealed no evidence of amyloid deposition in the heart or gastrointestinal tract, suggesting kidney-limited amyloidosis. Unfortunately, the patient died before therapy initiation because of accidental trauma (Fig. 3).

## Discussion

The patient was initially diagnosed with cryoglobulinemia-associated glomerulonephritis and MGRS. After 11 years of treatment with steroids and cryofiltration, the urinary protein level worsened relative to the cryoglobulin level, and a kidney biopsy was performed again, which revealed AL amyloidosis.

MPGN pattern is a glomerular injury characterized by mesangial proliferation and double-contour formation of the glomerular basement membrane. They can occur secondary to chronic infections, autoimmune diseases, or malignancies [4]. Cryoglobulins can also cause the MPGN pattern. Cryoglobulins are classified as type 1, which consists of monoclonal immunoglobulins, and type 2 and 3, which consist of polyclonal globulins [5]. Mixed cryoglobulinemia typically involves monoclonal IgM and polyclonal immunoglobulins and is most commonly associated with hepatitis C virus (HCV) infection. On the other hand, mixed cryoglobulinemia associated with monoclonal gammopathy by hematologic abnormalities is less common than that associated with infection. Kolopp-Sarda et al. reported that four of 50 patients with cryoglobulinemia had monoclonal IgG. They also reported that 40.8% of mixed cryoglobulinemia cases were secondary to infectious diseases (mainly HCV) [6].

In this case, the initial kidney biopsy demonstrated mesangial proliferation, endocapillary hypercellularity, double-contour formation of the glomerular basement membrane, hypocomplementemia, and circulating cryoglobulins, all of which are consistent with cryoglobulinemic glomerulonephritis. Although routine immunofluorescence did not detect immunoglobulin deposition, the possibility of a false negative could not be ruled out. Electron microscopy revealed organized microtubular deposits measuring approximately 35 nm in diameter, which fall within the reported ultrastructural spectrum of cryoglobulinemic glomerulonephritis. Furthermore, following treatment with corticosteroids and cryofiltration therapy, cryoglobulin levels decreased, and renal function and proteinuria improved. Collectively, these clinicopathological findings were most consistent with cryoglobulinemia-associated glomerulonephritis, although definitive immunoglobulin deposition could not be demonstrated. Conversely, the present case is atypical in that the cryoglobulin was composed of monoclonal and polyclonal IgG produced in the context of Monoclonal Gammopathy of Undetermined Significance, a rare cause of cryoglobulinemia. This rare combination of globulins may have caused the rare clinical course of sequential cryoglobulinemia-associated glomerulonephritis and AL amyloidosis.

AL amyloidosis is a known complication of monoclonal gammopathy with a reported incidence of 0.8% per year in patients with light chain monoclonal gammopathy [7]. Amyloidosis develops from monoclonal gammopathy through light chain misfolding [8]. Accumulation of misfolded proteins is gradual, but the addition of light chains to aggregated nuclei may lead to the rapid growth of amyloid fibrils. The risk factors for amyloidosis include high levels of monoclonal proteins, lambda light chain predominance, and abnormal free light chain ratio [9]. In this case, lambda light-chain predominance was observed.

MGRS encompasses a range of kidney lesions (e.g., AL amyloidosis and monoclonal immunoglobulin deposition disease), some of which are known to occur together [10–14]. This is thought to result from a shared pathogenic mechanism, namely, the presence of monoclonal immunoglobulins. In the present case, the patient sequentially developed two distinct MGRS-related lesions: cryoglobulinemia-associated glomerulonephritis and AL amyloidosis. Initially, the patient's cryoglobulins comprised monoclonal and polyclonal IgG, leading to the development of glomerulonephritis. As mentioned previously, cryoglobulins are typically composed of monoclonal IgM, but IgM-related AL amyloidosis accounts for only 4.4% of all amyloidosis cases [15]. The atypical composition of cryoglobulins in this case may have been associated with an increased risk of AL amyloidosis. However, IgM MGUS can lead to AL amyloidosis, similar to non-IgM MGUS [16]. This suggests that additional factors, such as environmental changes caused by steroid therapy or cryofiltration in the treatment of cryoglobulinemia, may be associated with amyloid formation.

Li et al. reported that repeated kidney biopsies could double the detection rate of MGRS in patients with MPGN patterns, suggesting that additional MGRS lesions may emerge over time [17]. Accordingly, clinicians should remain vigilant regarding the development of other MGRS subtypes in patients identified as having MGRS during the disease course. In this case, worsening proteinuria was observed without any corresponding increase in serum cryoglobulin levels or hypocomplementemia, raising the possibility of newly manifested AL amyloidosis. In addition, the κ/λ light chain ratio showed only mild skewing compared to the initial biopsy, and no amyloid deposition was identified in other organs, which made clinical suspicion of AL amyloidosis particularly challenging at that point. These findings highlight the importance of repeated kidney biopsies in patients with MGRS when there is recurrence or worsening of proteinuria, as this may indicate the development of a different MGRS lesion. Notably, AL amyloidosis differs from other MGRS subtypes in that effective evidence-based therapies, such as anti-CD38 monoclonal antibodies, are available [18]. Thus, accurate histological diagnosis is crucial for timely targeted treatment.

A limitation of this case is that paraffin immunofluorescence could not be performed on the initial kidney biopsy specimen because paraffin-embedded tissue was no longer available. Although routine immunofluorescence on frozen tissue is the standard method for detecting immune complex deposition, it has well-recognized limitations. In a subset of glomerulonephritis, immune deposits can be “masked” on frozen sections and only revealed by immunofluorescence on paraffin-embedded sections after antigen retrieval, leading to false-negative staining on routine immunofluorescence [19, 20]. In addition, comparative studies have demonstrated variable sensitivity of frozen versus paraffin immunofluorescence for different immunoglobulin classes, underscoring potential false-negative results in routine frozen immunofluorescence in certain cases [21].

## Conclusion

We present a case of AL amyloidosis that developed during the course of MGRS and initially presented as cryoglobulinemia-associated glomerulonephritis. Monoclonal immunoglobulins cause kidney lesions in MGRS. Therefore, when proteinuria worsens, a repeat kidney biopsy is essential to reassess the underlying pathology and guide appropriate treatment.
